# Data revolution, health status transformation and the role of artificial intelligence for health and pandemic preparedness in the African context

**DOI:** 10.1186/s12919-021-00228-1

**Published:** 2021-11-22

**Authors:** Sunny Ibeneme, Joseph Okeibunor, Derrick Muneene, Ishrat Husain, Pascoal Bento, Carol Gaju, Ba Housseynou, Moredreck Chibi, Humphrey Karamagi, Lindiwe Makubalo

**Affiliations:** 1 World Health Organization – Regional Office for Africa, Brazzaville, Congo; 2 World Health Organization – Headquarters, Geneva, Switzerland; 3 United State Agency for International Development, Washington, DC USA; 4 International Telecommunication Union, Addis Ababa, Ethiopia

**Keywords:** Africa, Artificial intelligence, Digital health, COVID-19, Healthcare innovation

## Abstract

**Background:**

Artificial Intelligence (AI) platforms, increasingly deployed in public health, utilize robust data systems as a critical component for health emergency preparedness. Yet, Africa faces numerous challenges in the availability, analyses, and use of data to inform health decision-making. Countries have limited access to their population data. Those with access, struggle to utilize these data for program improvements. Owing to the rapid growth of mobile phone ownership and use in the region, Africa is poised to leverage AI technologies to increase the adoption, access and use of data for health. To discuss and propose solutions for responsible development and adoption of innovations like AI in Africa, a virtual workshop was organized from the 21st to 24th June, 2021. This report highlights critical policy dimensions of strengthening digital health ecosystems by high-level policymakers, technical experts, academia, public and private sector partners.

**Method:**

The four days’ workshop focused on nine sessions, with each session focusing on three themes. Discussions during the sessions concentrated on public and private sectors, the academia and multilateral organizations’ deployment of AI. These discussions expanded participants’ understanding of AI, the opportunities and challenges that exist during adoption, including the future of AI for health in the African region. Approximately 250 participants attended the workshop, including countries representatives from ministries of Health, Information and Technology, Developmental Organizations, Private Sector, Academia and Research Institutions among others.

**Results:**

The workshop resolved that governments and relevant stakeholders should collaborate to ensure that AI and digital health receive critical attention. Government ownership and leadership were identified as critical for sustainable financing and effective scale-up of AI-enabled applications in Africa. Thus, government is to ensure that key recommendations from the workshop are implemented to improve health sector development in Africa.

**Conclusions:**

The AI workshop was a good forum to deliberate important issues regarding AI for health in the African context. It was concluded that there is a need to focus on vital priorities in deploying AI in Africa: Data protection, privacy and sharing protocols; training and creating platforms for researchers; funding and business models; developing frameworks for assessing and implementing AI; organizing forums and conferences on AI; and instituting regulations, governance and ethical guidelines for AI. There is a need to adopt a health systems approach in planning for AI to reduce inefficiencies, redundancies while increasing effectiveness in the use of AI. Thus, robust collaborations and partnerships among governments and various stakeholders were identified as key.

## Background

Artificial intelligence (AI) is a branch of computer science that uses algorithms defined by experts to recognize a problem or a task to be performed, analyze data, and make decisions, simulating human capacity [1]. Computerized decision support systems have been existent for decades. Evidence-based decision-making, monitoring of health status, tracking of expenditures and outputs for improving the efficiency of investments are hallmarks of successful health programs. In recent times, public health intelligence platforms, such as the World Health Organization’s Epidemic Intelligence from Open Sources (EIOS), utilize robust data systems as a critical component for health emergency preparedness. Within the development-partner community, prominent examples include PEPFAR and GAVI. Yet, Africa faces numerous challenges in the availability, analyses, and use of data to inform health decision-making. Enormous amounts of health data are now available, though countries utilize only a small portion of these data for program improvements. For instance, decision-makers could better and fully use demographic and health survey data at all levels. Health information systems are making large amounts of data available in several countries, but only a small portion of the data is being used to improve programs [2].

At the same time, the developed world is beginning to benefit from more advanced analyses and use of data. Technologies like AI have the potential to offer new insights and tools to improve clinical decision making, predictive analytics for health emergency preparedness, mitigate workforce shortages, tailor programs targeting areas of greatest need, enhance forecasting of disease outbreaks, and bring efficiencies to health service delivery. Realizing the potential of digital health and AI to achieve the development goals will require collaboratively addressing multiple challenges, including strengthening underlying digital infrastructure and digital health systems, working to improve data quality, responsible management and sharing of available data, and eventually trust and utilization of data to inform strategic planning [1, 2].

Africa is in some ways poised to make use of these technologies through the rapid growth of mobile phone ownership and use. However, there remain significant gaps in the digital health ecosystems that will shape its utilization. Realizing digital technology’s full potential, including AI, requires attention to policy and the regulatory environment, system infrastructure, sustainable financing, technical safeguards and workforce capacity, and diversity of the stakeholders involved in digital health and technology innovation. While Africa must not be left out of new advances, it is important to approach innovations thoughtfully including its applications to preparedness.

Globally, the COVID-19 pandemic has triggered an unprecedented demand for digital technology solutions in screening populations, tracking infections, and minimizing direct human contact. The use of different technologies at every step has brought efficiencies in monitoring and contact tracing, testing, and case management [2, 3]. Most importantly, technology has played and continues to play a vital role in protecting medical personnel by limiting direct contact with patients, disinfecting controlled environments, and disseminating public health and emergency messages [4]. By leveraging digital technology in Africa in the health sector, different health crises can be managed better, and health care systems strengthened through more effective and efficient digital mechanisms.

The AI workshop brought together high-level policymakers, technical experts, and public and private sector innovators to discuss critical policy dimensions of strengthening digital health ecosystems and lay the foundation for responsibly developing and adopting innovations like AI among African health systems. The workshop took place over 4 days. The first day highlighted existing AI-based applications with an in-depth showcase of the development processes. On the second day, policy issues that impact the application and use of digital solutions in countries were highlighted and recommendations made. Country experiences were shared on the third day including frameworks for involving the private sector and donors. The last day was focused on defining the next steps and action points on how to address issues identified in the first 3 days.

This workshop was jointly organized by the World Health Organization – Regional Office for Africa (WHO/AFRO) and the International Telecommunication Union (ITU), with sponsorship from the United State Agency for International Development (USAID). Other agencies that contributed in the organization of the workshop include but not limited to AfDB, UNICEF, UNESCO, UNECA, JICA, EAC/EAHRC, Digital Square, DIAL, HHA, I-DAIR, and AUC, among others. Participants were mainly countries’ representatives from the Ministries of Health, Finance, Information and Technology, development organizations, the private sector, academia, researchers, and Information Technology (IT) experts among others. On the average, about 250 participants attended the workshop. Notable attendees were the African Regional Directors for the WHO/AFRO, ITU, USAID, and a representative of the Honorable Minister of Health, Republic of Togo. All invited speakers represented institutions working with digital technology including implementing partners, academia, developmental agencies, government and other IT experts who provided key experiential insights into the workshop technical sessions.

### The WHO AFRO agenda for digital health

This session revolved around three thematic focal areas including: setting the scene, which provided digital health context in the African region; global perspectives, which highlighted WHO’s global digital health initiatives, and Regional views, which introduced WHO’s digital health activities in the Africa region. The session drew on lessons learned in the past decade in digital health at the global, regional and national levels. Keynote presenter summarized data elements from the Global Observatory on eHealth highlighting critical issues as it relates to the digital infrastructure landscape of countries in the region. This triggered discussions and reflections on the future of digital technology in health; and how these can be used to advance discussions on Universal Health Coverage (UHC), the WHO 13th General Programme of work and the health Sustainable Development Goals (SDGs). Opportunities and challenges for digital health in the wake of COVID-19 were highlighted including the future of digital health in Africa.

Keynote presenter gave a high-level summary of WHO AFRO’s commitments to promote good health, keep the world safe, and serve the vulnerable. The presenter maintained that WHO established an impact framework to help streamline its efforts in these focal areas. At the bottom of the pyramid are 46 outcomes indicators and milestones which are aligned with the SDGs. These indicators are in line with WHO approaches towards UHC, health emergencies, and healthier populations; and corroborate WHO’s triple billion targets which targets to increase healthy life expectancies [5].

In addition to establishing an impact framework, WHO AFRO also established an operational framework that includes health and innovation as parts of its integrated service delivery in primary health care, research, development and innovations. The framework has informed the development of four resolutions since 2005. These resolutions include the 2005 WHO resolutions on eHealth, followed by two resolutions in 2013– the Global eHealth standardization and interoperability resolution, and The regional WHO/AFRO resolution on eHealth. The regional resolution, also known as resolution AFR/RC60/R3, underscores priorities such as the need for improved political commitment, the need to have national eHealth champions, the need for multi-sectoral coordination for digital health, and the need for ICT infrastructure support, and ICT curriculum institutionalization in learning environments. Since the adoption of these resolutions, WHO/AFRO has focused its efforts on creating an enabling environment to include technology in the health sector. The fourth resolution was the Global WHO resolution on digital health in 2018 [6].

Speaking on these resolutions, the keynote presenter underlined the cross-industry and cross-society nature of digital transformation in health in the twentieth century. The presenter reiterated the importance of collaboration and co-creation in ensuring quality digital solutions and the willingness to adopt proposed changes. Keynote presenter maintained that the WHO’s Headquarters has reinforced its commitment to support Regional Offices and Member States to establish a unit to support collaboration and capacity building in digital health. The digital landscape of the region was appraised with respect to innovations, digital products, operational changes, digital health tools and guidelines. In addition, the keynoter presenter highlighted the WHO Global Strategy on Digital Health as the critical resource to guide a people-centered approach to digital transformation in the health sector [6].

Speaking on the contributions of the private sector, the keynote presenter started by enumerating the disparities in the health workforces in Africa compared to other continents. The databank from the World Bank showed that 31 out of 54 countries in Africa have less than 10 Doctors per 100,000 people, compared to the 240 per 100,000 people in Germany and 370 doctors per 100,000 people in Italy [3]. These gaps illustrated the urgency of frontier technology to extend services to more people in Africa. A study by GSMA highlights the opportunities for technology in Africa. The report shows that 475 million people in Sub-Saharan Africa will have access to mobile internet by 2025 [3]. This indicates a massive opportunity for digital health. However, digital health startups face challenges in Africa including inadequate funding, lack of data culture, insufficient digital infrastructure, stakeholders not adopting local solutions and lack of appropriate regulatory frameworks and standards among others.

During the question and answer session that followed keynote presentation, participants gave further insights on the Regional perspective on digital health. According to statistics from UNESCO database, 65% of African countries currently deploy data in education, health, agriculture, environment, and industrial sectors [3]. In addition, the emergence of AI research capacities and institutions in Africa has attracted investments from larger global technology giants. Examples of such investments are Google investments in Ghana and IBM in South Africa [4]. Participants maintained that Africa needs a continental strategy on AI and recommend the creation of regional sectors to harmonize the regulation and ethics on the use of AI for health in the region. This session concluded by highlighting progresses made in the deployment of DH technologies in support of service delivery in the African Region despite persisting challenges. Key recommendations include: Develop a continental strategy for AI; Create regional centers in applied research for the use of AI in key sectors including health, education, agriculture, and environment; Encourage the development of innovative and broadband networks on the continent; Harmonize regulation of the use of AI for health in the continent; Foster and encourage public-private-partnerships in the AI field among African economies.

### COVID-19 and digital health opportunities

Following conclusions from session 1, this session focused on the use of digital technologies in surmounting COVID-19 pandemic. It demonstrated critical digital health interventions in the African region that are available and scalable for COVID-19 response. Topical issues discussed include: digital governance, sustainability, and the next steps of health sector digitization in the Region. In the wake of COVID-19, health systems around the world have been under immense pressure. Thus, countries worldwide including African countries have taken vital steps in using digital tools to mitigate the effects of COVID-19 outbreak. Topical presentations discussed in this session include: AI for Health Programs (COVID-19)– opportunities and perspectives; Telemedicine, health data governance, electronic health record systems including patient reminders.

Keynote presenter enumerated existing projects that enhanced health care professionals’ capacity to provide quality care and identify specific capacity needs. The presentation focused on identifying roadblocks that impede the deployment of AI among health programs in Africa. Keynote presenter maintained that the University of Geneva has active projects in francophone parts of West Africa, specifically Senegal, where the team has deployed an AI application to identify optometry-related illnesses. The presentation also illustrated the challenges faced in implementing AI applications. Key challenges listed include: lack of quality data to train algorithms, the impossibility of questioning an algorithm when questions arise. Keynote presenter concluded by recommending that researchers make algorithms understandable, educate professionals and the public about AI and its potentials. The presenter reminded individuals who practice medicine and AI to remember the principles of benevolence and vigilance. These two principles urge practitioners to do no harm and perform regular verifications of algorithm performances respectively.

The session continued with another topic expert speaking on Telemedicine. Keynote presenter led the audience through the evolution of telemedicine. Telemedicine was defined as, “The provision of health services, where distance is a critical factor, using information and communication technologies to exchange valid information for diagnosis, treatment and disease and injury prevention, research and evaluation, and for the continuing education of health professionals, intending to promote the health of individuals and their communities [7] The presentation also promoted the advantages of telemedicine, pointing out that the inclusion of technology has made access to healthcare very easy. The presentation covered the various national and international digital health initiatives where African ministries of health, researchers, and physicians have contributed to the progress of telemedicine. However, the keynote presenter called for the need for increased ethical standards in the evolving field of telemedicine, recommending the adoption of the World Medical Association Declaration on telemedicine [8]. Finally, a demonstration was made using use-cases to highlight the critical role of telemedicine in sustaining health systems during the COVID-19 pandemic. This facilitated virtual consultations between care providers and patients.

During the question and answer session that followed keynote presentation, participants sought further insights on the procedural needs identified during the COVID-19 pandemic for digital tools within the African context. Responding to these concerns; keynote presenter listed existing and prospective technology-based solutions and data analytics mechanisms that facilitate the management of the pandemic responses in the region. The presenter maintained that inherent systemic challenges have continued to impede the effective use of digital technology for health. These challenges include but not limited to interoperability issues, absence of a comprehensive digital platform that can guide solutions, and lack of access to real-time patient data to help recommend approaches. The session concluded by recommending WHO/AFRO and ITU to continue to support African health systems for the SDGs by integrating emerging innovative technologies into existing systems.

### Artificial intelligence and health systems interventions

This session discussed the role of health systems strengthening through AI-enabled digital mechanisms. Keynote presenter started by defining key AI concepts including: 3-D, Blockchain, Smart Infrastructure, Biomedical Engineering, and Bioinformatics, and summarized mechanisms through which these innovations can empower patients’ access and utilization of health services. Use-cases were used to demonstrate how these are being operationalized.

Keynote presenter reiterated the definition of the fourth industrial revolution technologies, comparing AI to the sub-fields of Machine Learning and Deep Learning and other related types of Machine Learning algorithms. While AI was defined as any technique that enables computers to mimic human behaviour, Machine Learning was defined as a subset of AI techniques that use statistical methods to enable machines to improve with experience. Keynote presenter noted Deep Learning to be a subset of Machine Learning which makes the computation of multi-layer neural networks feasible.

Further in the session, the keynote presenter summarized a case of how machine learning could be leveraged to understand and support human resource for health (HRH) activities. The project focused on supporting South Africa’s Human Resources for Health Strategy 2030. The goal was to establish processes for effective workforce planning which aligned with national needs. The project utilized existing data to observe a difference of an estimated 100,000 HRH between the public and private sector of South Africa’s health workforce. This study targeted to mitigate attrition faced by public sector HRH during the COVID-19 pandemic, and explored mechanisms to cushion the effects of the pandemic on the health workforce. The presentation concluded by emphasizing the fact that these projects are setting the groundwork for the broader adoption of ML and AI in the health sector in Africa. Keynote presenter also urged the collaboration of partners from various sectors to understand health systems problems, and leverage technology to develop solutions while addressing ethical and data protection challenges collectively.

During the question and answer session that followed keynote presentation, participants reiterated the need for purposeful, concerted approaches to operationalize AI strategies in the Region; and the importance of leveraging contributions from various stakeholders. They also acknowledged that ITU’s position is critical, as connectivity plays an essential role in expanding digital health. Participants expressed keen interest in discussing data privacy and mechanisms to manage data challenges in the Region. They sought to understand the mechanisms that current digital health start-ups employ to address the issues of data privacy and confidentiality. A consensus was reached on data-sharing approaches including data research methodologies and development and the importance of anonymizing data. A recurring phenomenon during this session was why the healthcare industry appears less proactive than other industries in adopting AI. Keynote presenter addressed this issue by pointing out that the healthcare industry deals directly with human life. Therefore, relevant checks, regulations, and policies need to be in place to ensure proper accountability and mitigate any form of anticipated challenges regarding AI use for health. This session concluded with key recommendations for African governments including: Invest in human capacity and development; Establish the Africa Artificial Intelligence Institute; Create, review and amend policy and legislation; Establish a platform to advance the manufacturing of new materials; Secure and avail data to enable innovation; Incentivize future industries and build a fourth industrial revolution of infrastructure.

### Artificial intelligence for health programmes in the African region

This session showcased the various AI initiatives in the African region and explored the foundations for which these technologies were being established, while drawing best practices from the global community. Keynote presenter maintained that WHO/AFRO and ITU have continued to coordinate and support the deployment of government-owned AI digital solutions in the Region, and called other development partners to work collaboratively with them on similar initiatives.

Lessons from Rwanda were shared among participants which highlights a vision of how AI can help improve healthcare quality, especially in the context of COVID-19 pandemic. Keynote presenter maintained that the preparedness and systems that were set up were all based on the lessons of the Ebola outbreak. The government of Rwanda developed new strategies and harnessed existing tools and technologies to drive country preparedness. Topical technologies like drones and robots were introduced to support country preparedness and responses. Robots were used to measure patients’ temperature, and ensure individuals continuously wear masks at public places like the airport. It also enabled healthcare professionals to communicate virtually with patients, as well as coordinate health surveillance mechanisms.

The Lusophone experience was also shared by the keynote presenter. Experiences from Guinea Bissau as a representative of Lusophone countries highlighted the strengths from partnerships. Keynote presenter maintained that the partnership with the government of India had led to the installation of the Telemedicine Center in Guinea Bissau since 2010. This has provided such services as: ultrasound, radiography, videoconference, and technician services among others. Thus, with such systems at the Telemedicine Center, there has been a decrease in medical tourism among the citizenry. It was also stated that the center had also helped in disseminating and providing health education for preventive and diagnostic measures, while reducing the transfer of patients to referral facilities.

Different countries’ experiences showed varied perspectives on AI for health in Africa. However, they underscored common strengths and weaknesses, which include improving health service quality and reducing the burden of overcrowding and bed shortages at health facilities. Thus, AI is poised to assist countries to improve the quality of service delivery through innovation using telemedicine-assisted approaches. The session concluded by emphasizing the need to deploy new technologies and share lessons and experiences among countries to help improve healthcare in Africa. Recommendations from the session were that: AI tools for health could facilitate information sharing, diagnostics and monitoring during the prevailing COVID-19 pandemic, and have opportunities to help improve health systems.

### Artificial intelligence for good; current situation of AI use in healthcare around the world

The global community has been instrumental in providing lessons learned in digital health solutions implementation. A key goal of digital solutions is to enable better health outcomes and fast-track actualization of UHC and health SDGs as well as other internationally agreed goals and targets. This session explored the proliferation of AI solutions globally and discussed their maturity.

The inclusion of AI in health is a clear commitment to support equitable access to quality health services as countries work towards achieving UHC. Keynote presenters provided in-depth guidance on the WHO global strategy on Digital Health 2020–2025. The strategy highlighted four strategic objectives including: Promote global collaboration and advance the transfer of knowledge on digital health; Advance the implementation of national digital health strategies; Strengthen governance for digital health at global, regional and national levels; and Advocate people-centered health systems that are enabled by digital health [6]. Keynote presenter maintained that working with these objectives will improve health for everyone by making affordable and scalable digital health solutions available among populations [9]. According to studies, research and publications on AI for health is increasing [10]. However, many challenges exist, but improving the technical infrastructure, data management, and cost of digital platforms and regulations can help to address topical challenges. A joint effort from experts in diverse fields can help address identified challenges.

Keynote presenter appraised the importance of AI in the treatment of diabetic retinopathy and cervical cancer; stating that since 2018 there has been a joint focused group between WHO and ITU on AI for Health (AI4H) activities [11, 12]; and there are other related inter-agency collaborations on AI. WHO and ITU establish benchmarking processes for artificial intelligence in health [12], and have established five working groups dedicated to various thematic aspects of AI for health. These groups include: Ethical considerations on AI4H (WG-Ethics); Regulatory considerations on AI4H (WG-RC); Clinical evaluation of AI4H (WG-CE); and AI solution Assessment Methods, Data (WG-DASH); and AI solution Handling. These working groups create best practices, establish processes and related policies, define ways to successfully benchmark AI for health algorithms, and create reference documents as necessary [12, 13].

Keynote presenter gave a high-level summary of AI maturity in health self-assessment tools. Landscape analysis shows that AI improves population health, pre-clinical research and clinical trials, clinical care pathways, patient-facing solutions, and optimization of health operations among others. Keynote presenter enumerated six-step maturity roadmaps for AI, prioritizing the following areas: people and workforce; data and technology; governance and regulations; design and processes; partnerships and stakeholders; and business models. Participants were encouraged to explore the free AI maturity in Health self-assessment tool [14]. Keynote presenter highlighted the importance of AI that can empower patients to manage their own health; health providers to deliver better healthcare; health managers to transform health systems; and governments to improve population health.

Another presentation was made on AI and Universal Health Coverage. Keynote presenter highlighted the challenges affecting UHC in developing countries including uneven distribution of health professionals among Sub-Saharan African countries. The presenter discussed critical ways AI can help address the uneven distribution of healthcare professionals for specialized care consultations and preventive care in Africa. Using Senegal as a case-study, the keynote presenter enumerated the collaborations between the Ministry of Health, ITU, and WHO on the Project “Equal Access to Ophthalmologists”. This project using the AI platform (Bogou) helps in the timely detection and management of diabetic retinopathy and connects peripheral health centers to specialists [15]. The session concluded that AI is an asset for UHC and has opportunities to reduce the cost of care, improve the quality of care, and facilitate inclusiveness and scale-ups.

During the question and answer session that followed keynote presentation, participants highlighted notable challenges that impede the use of AI tools for achieving UHC, and made some suggestions for considerations including: a need to create a technical infrastructure for data storage, develop algorithms that assist in rapid diagnostics, and identify and address risk factors that hinders patients from seeking healthcare on time.

### Regulatory issues in artificial intelligence for health

Digital technologies including AI applications play a critical role in strengthening patient safety, infection prevention and control to prevent harm caused by infection to patients and health workers. The session discussed crucial issues related to AI governance, ethics, privacy, safety, human rights, equity, capacity building, regulations, and data governance.

The first presentation was on AI governance at the national level: A framework for action. Keynote presenter maintained that the COVID-19 crisis had refocused attention on health systems’ investments, resilience and digital opportunity. It was stated that digital health offers patient-centric and cost-effective ways to reinvent the delivery of health services. Amidst these opportunities lies the challenges of gaps in infrastructure, human capacity, and governance. Accordingly, AI governance must be value-based, collaborative, agile, distributed and end-to-end. Keynote presenter summarized a novel governance mechanism for health AI that focuses on building stakeholders’ engagement tools, robust governance frameworks and innovative exchange architecture [16]. Values and principles help build a culture of human-centered technology development and governance. Keynote presenter shared core values that can drive AI governance in Health. These core values are human-centeredness and human agency, diversity, trustworthiness, fairness, collaborations, independence and neutrality; transparency, innovativeness and inclusivity among others.

Further in the presentation, keynote presenter indicated that Ethical AI is artificial intelligence that adheres to well-defined ethical guidelines regarding fundamental values, including individual rights, privacy, non-discrimination, and non-manipulation. The presenter explained that representativeness in data, its explainability and inherent biases are some of the reasons for ethical challenges. Regarding biases, numerous societal biases currently influence the use of data including systems ownership, ethics in AI, human rights, and equity among others. There is a need to implement fairness and equity at the algorithm level. Keynote presenter maintained that current AI ethics are relatively undefined; however, AI cannot be discussed without ethical considerations.

Another presentation was made on AI Regulations in Health and Data Governance. Keynote presenter highlighted ongoing activities in various African countries on AI. The presenter listed countries that were currently developing national AI strategies to include Mauritius, Egypt, Zambia, Tunisia and Botswana. Countries like Ghana, Kenya, Nigeria, Senegal, Sierra Leone, South Africa, Tunisia, Uganda, Zambia, and Zimbabwe were listed to be promulgating national AI laws and regulations. Rwanda, Kenya, Tunisia, South Africa, Uganda, Tanzania, and Cameroon had started initiating public sector reforms with AI. Some African governments were noted to have approved and formed AI centers and programmes to promote AI knowledge acquisition and development. These governments include Egypt, South Africa, Rwanda, Cameroon, Morocco, Senegal, Lesotho, Namibia, Ethiopia. However, countries like Ghana, Tunisia, Rwanda, Uganda, Zimbabwe, Ethiopia, Mali, Egypt, Malawi, Namibia, Côte d’Ivoire, Kenya, Nigeria, and Senegal have started building strategic partnerships, which is vital for AI adoption in countries. Egypt, Kenya, Mauritius, Nigeria, South Africa, Tunisia, and Uganda have established national AI agencies, task forces and commissions [17]. These reports showed that African economies have started legitimizing AI. The Keynote presenter maintained that AI uses vast amounts of data. This directly conflicts with the preservation of privacy, hence the need for governance. The established governance structures would focus on understanding the implications of AI, its rewards and its risks. The deliberations concluded with a call for WHO and ITU to collaborate to establish governance and regulatory mechanisms to guide AI innovations in Africa. In addition, an increase in the use and quality of the internet on the continent will be helpful. This will enable the adoption and use of AI for health at the country level, especially among low- and middle-income countries.

During the question and answer session that followed keynote presentation, participants enumerated inherent potentials embedded in the use of AI for Health. This session concluded that AI has huge potentials with many risks. Hence, good governance and regulations must be encouraged through innovation to manage the risks.

### Artificial intelligence for health financing

This session seeks to provide a framework for costing AI health technologies. It highlighted key cost considerations and business models needed to responsibly implement AI-driven health interventions. The session also provided a review of reference cases of AI-driven health technologies currently under development in Sub-Saharan Africa through partnerships with the private sector.

Innovative business and management models are vital for scaling and building sustainable AI-driven solutions for healthcare [18]. Keynote presenter summarized frameworks for identifying and assessing the costs of AI-driven health technologies. Keynote presenter maintained that while costing AI interventions, that the primary interventions involved are the system design and development, rollout, recurrent digital infrastructure, and AI management. The presenter highlighted how relevant efficiency and designing cost interventions are, when it comes to AI projects. This was demonstrated by using a reference case of the Burkina Faso Healthcare Worker AI generated by Terre des Hommes IeDA [19]. This model informed robust demand generation in-view of budget allocations for Monitoring & Evaluation, and demonstrates the relevance of product proof-of-concept for improved efficiency and effectiveness. Thus, stakeholders are to consider the price of digital infrastructure in their respective budgets as part of the initial project conceptualization and development.

The role of the private sector was also discussed. Keynote presenter maintained that the private sector plays a key role when it comes to financing AI health technologies. Purposeful partnerships with public and private sectors are keen to driving innovation on the African continent. Keynote presenter cited that the Phillips as a company engages in impact finance by co-funding a collaboration with the Dutch Development Bank. The co-funding partnerships fund proof-of-concept projects in Africa, assess investment opportunities in specialized primary care impact funds in Africa, and cooperate with impact funders and investors to scale new and innovative healthcare delivery models. Phillips provides access to quality care in underserved communities by strengthening community and primary health care systems [20]. It offers a HealthSuite benefit of collecting and storing patients’ information and data with affordable technologies.

Further in the presentation, keynote presenter speaking on AI business models, defined business models as an organization’s core strategy for profitably doing business using AI [21]. Keynote presenter maintained that considering how fragile and vulnerable health systems are, it is relevant to create strategic investment to increase the impact of health interventions. The need to identify approaches to AI business models to ensure sustainable AI adoption was re-emphasized. In developing AI business models, there is the need to consider co-development arrangement with customers, at-risk pricing, direct-to-consumers, data curation and preparation of data libraries, investment ahead of regulatory and public expectations, and SaaS models among others [22]. The four principal models to consider include the following: Value Model – the business model must benefit all stakeholders; Financial Model – there is a need to consider federation learning models instead of centralized models. This allows for a split of costs and fees among stakeholders; Technological Model – foster projects acceptance and success by creating human loops where necessary; and Distribution Model – ensure continuous partnerships, marketing, and sales for sustainability [21, 22].

During the question and answer session that followed keynote presentation, participants raised concerns regarding the proliferation of siloed AI tools among countries and their consequences. They called on the WHO/AFRO and ITU to seek ways to develop unified business models for adoption and use among African economies. The session concluded with key recommendations that costing and evaluation of data can help understand AI’s return-on-investment (ROI) in terms of health-savings and cost-savings. Governments should partner and provide an enabling environment for private sector investment in AI health technologies. In addition, partners should adopt strategic business models to ensure sustainability of AI projects.

### Predictive analytics for health

This section focused on how AI through a well-coordinated data ecosystem can be used to predict health emergencies. Keynote presenter summarized how AI-driven predictions can improve health systems’ efficiency. The presenter maintained that AI technologies have many potentials to improve healthcare in Africa. However, there are challenges in deploying AI, such as biased data and algorithms; others are bias in digitized, representative and accessible data, as well as inadequate infrastructure among others. A broad distinction was made between AI and traditional predictive analytics; owing to the growing amount of data which makes it difficult to optimally analyze data using standard methods. AI-based predictive analytics can analyze both historical and real-time data. It can also analyze important, unstructured information stored in disparate data systems and platforms. AI-driven predictions offer a holistic assessment of a given situation and can be used to strengthen resource allocation processes [23]. Keynote presenter maintained that emerging technologies like AI could also help to improve health worker performance. The case of the IeDA project was used to explain how algorithms identify irregularities in consultation data, and this is being used to precisely predict in real-time the most common clinical errors and provide instant feedback [19].

Keynote presenter also explained how various collaborative partnerships among researchers and young entrepreneurs have continued to solve global health challenges. Through collaborative mechanisms, digital technologies and AI are playing an essential role in sampling, analyzing and presenting data from multi-disciplinary knowledge and diverse data sources across the human-animal-environment interface. The presenter explained how INSPIRE PEACH, an East African platform project, has proposed to develop the critical elements of a coordinated Pan-African COVID-19 data ecosystem. Currently, the database from Kenya and Malawi are being used to help COVID-19 harmonization in these countries. This is helping in transforming data into information for policy-making as part of COVID-19 response.

During the question and answer session that followed keynote presentation, participants urged WHO and ITU to work concertedly to establish mechanisms that could foster cohesive synergy between data analysts and users to ensure regional databases deliver policy and programmatic expectations. The session concluded with recommendations that AI-based predictions should support decision-making processes by increasing the accuracy of future outcomes, improve data usability for targeted resource allocation, and enable proactive health systems. It was established that collaborative partnerships among stakeholders have opportunities to improve AI-enabled service delivery in Africa. Thus, data and AI can improve policy issues by providing scientific evidence for policymaking which could leaders to plan effectively. African governments are encouraged to bring all stakeholders together for continuous engagements and deliberations on AI-enabled data analytics and predictions.

### Collaboration and partnerships in the digital health ecosystem

This session discussed frontier technologies and how various sectors play crucial roles in the digital health ecosystem among African economies. Keynote presenter maintained that there is no single definition of frontier technologies. However, they are generally understood as new and rapidly evolving technologies that take advantage of digitization and connectivity. Based on the country readiness index, most of the least ready countries to frontier technologies are in sub-Saharan Africa [24]. Despite low resources and capabilities, frontier technologies can increase Africa’s productivity and improve livelihoods. For example, AI-enabled tools used by TrueSpec Africa in West and Central Africa to detect fake drugs; robots used in Rwanda as part of COVID-19 AI response innovations. Keynote presenter highlighted several challenges that affect countries including: low technology and innovation capabilities, poor diversification of technology, weak financing mechanisms, intellectual property rights, and dearth in technology transfer among others.

This session also gave a discussion on the role of the private sector. Keynote presenter explained the critical role the private sector plays in Africa in various market spaces. The digital market in sub-Saharan Africa has enormous growth potentials. The mobile industry contributes 8.6% of the GDP in 2018 and is expected to increase to 9.1% by 2025. Since 2018, there are 239 million mobile internet users across Africa, and it is estimated to increase to 483 million users by 2025 [4]. Data from stakeholders’ assessment of Africa’s readiness to take up technologies conducted by AfDB showed that those that are ‘willing and ready’ for AI use were 35%, ‘somewhat willing’ were 35% and ‘not ready’ were 30% [1, 4]. Thus, cooperation is needed to improve digital infra- and e-government to improve AI on the continent. The public-private-partnership is critical to enhance rural broadband deployments and internet access, internet access infrastructure, and e-government policy and platforms among others.

The academia was also identified as a critical element of this conversation as it has a role to play in improving digital health. Throughout this pandemic, innovative technologies like AI-enabled tools have answered the call for a new form of public health that illustrates opportunities for enhanced agility, scale, and responsiveness [25]. The academia has a role to develop frameworks, models and theories for the development of AI, and address AI challenges such as ethics, governance and regulations. Through evidence-based recommendations to policy and intensifying research on the creation and analysis of AI-based models, the academia can support public health to improve AI in the region. Keynote presenter summarized that AI has great potentials to enhance public health by using AI agents to simulate early warnings and alerts; contact tracing; scenario prediction; cure, vaccines, treatment novelties; data dashboards and social media information warfare controls among others.

During the question and answer session that followed keynote presentations, participants expressed interests in the mechanisms and the cost of strengthening human resource capital in AI for health. They called for a strengthened collaboration between governments, the private sector, the academia and related research institutions in institutionalizing digital health, and AI to improve public health. The session concluded with recommendations that countries should be prepared to use, adopt and adapt to the emerging technological revolution.

## Conclusions

On the last day of the workshop, participants were grouped along language lines to deliberate on pertinent issues regarding workshop objectives. Participants were grouped into three working groups based on the WHO/AFRO official languages including English, French and Portuguese languages. Country representatives from the WHO/AFRO, Member States, ITU and other stakeholders shared their reflections on the workshop, as well their expectations moving forward on AI for health in Africa.

Participants expressed appreciations for the quality presentations with many learning experiences and opportunities from the workshop. They noted the timeliness of the workshop and emphasized the need to sustain the momentum of sensitization in the region to ensure wide adoption of AI in Africa. Participants reiterated the need for the WHO and ITU to develop a roadmap with a focus on generating and owning data, and expanding research capacity on AI. They also called for sustained collaborations with all stakeholders and professionals on the continent to continue discussions and dialogues on AI for health in the region.

Participants highlighted many areas of priorities on AI in the region including data protection, privacy and sharing protocols; training and creating platforms for scientists and researchers; developing funding and business models for assessing and implementing AI in the Region; organizing forums and conferences on AI; and developing guidelines and regulations for AI ethics among others. In addition, participants proposed that countries prioritize capacity building to promote and prepare for the adoption of digital health. It was recommended that various partners provide training and establish forums among Member States for capacity development on AI for health. Big technology partners were encouraged to support researchers in AI, Business Intelligence and Machine Learning by easing access to data, especially anonymized health microdata.

Furthermore, there is a need to adopt a health systems approach in planning for AI to reduce inefficiencies, redundancies and fragmentation while increasing effectiveness in the use of AI for health systems strengthening. Resistance to change remains an influencing factor for the slow implementation of AI in Africa’s healthcare industry. This was discussed widely and topical mitigating measures identified and include but not limited to: consistent commitment to work among those willing to work on AI technologies, government commitment and investment, and continuous funding opportunities from internal and external sources. Unanimously, it was agreed that there should be at least one pilot AI project in one African country for other countries to emulate.

### Recommendations

We call on the Ministries in charge of Health and ICT of all Member States in Africa and all stakeholders to ensure that the key recommendations of this workshop are implemented. Specifically, we call on the health sector leaderships of all African countries to provide the required leadership for DH and AI in their nations. We also call on governments and all stakeholders to come together to help ensure AI and digital health receive critical attention in the development of the healthcare industry in Africa. There are recommendations explicitly directed to WHO, ITU and Member States. They include:

#### WHO/AFRO

It was recommended that WHO/AFRO establish an AI Data Governance in the African region; Develop AI road map for the African Region; Create a periodic forum for scientists, researchers and other related professionals to get awareness and discuss AI for health issues. Establish a platform for the exchange of ideas and experiences on AI among Member States; Empower national-level organizations responsible for digital health to support countries in implementing programs; Establish in each country a national program to support the development of digital healthcare to drive national digital health strategy.

#### ITU

It was recommended that ITU continue to ensure the exchange of experiences and share knowledge to support the development of a digital health framework for African countries; Strengthen digital infrastructure and improve access to meaningful connectivity; Support the implementation of digital health strategies and build capacity for digital health focal points in the Africa Region; Facilitate the increased use of AI and other digital technologies in the health sector to advance digital transformation in the Africa region; Leverage and strengthen multi-stakeholder partnerships for sustainable adoption of digital health and resilience of the health systems in Africa.

#### Member states

At the national level, it was recommended that Member States develop a country roadmap focusing on the regional roadmap; Assess the readiness of country AI maturity level; Prioritize capacity building and infrastructure; Establish financing mechanisms and allocate budget lines for the computerization of health facilities and digital transformation as part of national health systems’ reforms; Organize an exchange meetings of national clinical associations and private practices on digital health.

## Data Availability

The materials and information used in developing this manuscript were obtained from the final report of the AI workshop report, and is available on request from the corresponding author.

## Abbreviations

AfBD: Africa Development Bank
AI: Artificial Intelligence
APHRC: Africa Population Health Research Center
DH: Digital Health
GAVI: Global Alliance for Vaccines and Immunization
ICT: Communication Informatics Technology
I-DAIR: Digital Health & AI Research Collaborative
INSPIRE PEACH: Implementation Network for Sharing Population Information Research Entities Platform for Evaluation and Analysis of COVID-19 Harmonized data
ITU: International Telecommunication Union
MoH: Ministry of Health
UHC: Universal Health Coverage
WHO: World Health Organization
WHO/AFRO: World Health Organization Regional Office for Africa
